# Optimization of the Energy Consumption of Depth Tracking Control Based on Model Predictive Control for Autonomous Underwater Vehicles

**DOI:** 10.3390/s19010162

**Published:** 2019-01-04

**Authors:** Feng Yao, Chao Yang, Mingjun Zhang, Yujia Wang

**Affiliations:** College of Mechanical and Electrical Engineering, Harbin Engineering University, Nangang District, Harbin 150001, China; yangchao@hrbeu.edu.cn (C.Y.); zhangmingjun@hrbeu.edu.cn (M.Z.); wangyujia@hrbeu.edu.cn (Y.W.)

**Keywords:** autonomous underwater vehicles, model predictive control, trajectory tracking, energy consumption optimization, cost function

## Abstract

For long-term missions in complex seas, the onboard energy resources of autonomous underwater vehicles (AUVs) are limited. Thus, energy consumption reduction is an important aspect of the study of AUVs. This paper addresses energy consumption reduction using model predictive control (MPC) based on the state space model of AUVs for trajectory tracking control. Unlike the previous approaches, which use a cost function that consists of quadratic deviations of the predicted controlled output from the reference trajectory and quadratic input changes, a term of quadratic energy (i.e., quadratic input) is introduced into the cost function in this paper. Then, the MPC control law with the new cost function is constructed, and an analysis on the effect of the quadratic energy term on the stability is given. Finally, simulation results for depth tracking control are given to demonstrate the feasibility and effectiveness of the improved MPC on energy consumption optimization for AUVs.

## 1. Introduction

Autonomous underwater vehicles (AUVs) are a unique solution for underwater missions, including resource exploration, target search, submarine pipeline tracking, environmental monitoring, and submerged floating operations [1,2,3]. AUVs’ motion control is a significant part that helps them to complete their tasks and confront many difficulties, such as model uncertainties [4], limited resources [5], and constraints [6]. The limited onboard energy resource is one of the key factors affecting long-term AUV detection, cruise, operation, and other missions in the complex marine environment [5,7]. One of the most perceivable trends for marine robotics is the development of energy efficiency [8]. Therefore, energy consumption optimization is an important part of AUVs’ ability to complete their tasks [9,10].

An energy consumption reduction for underwater vehicles can be achieved by a variable buoyancy system (VBS) and improvement of the control method. A VBS is one effective approach to reduce the energy consumption that is widespread in the gliders of AUVs that need a large depth change [5]. However, it cannot be implemented in AUVs without a VBS. Unlike the depth control for VBS-equipped AUVs in which the buoyancy can be adjusted, researchers can achieve the goal of an energy consumption reduction by optimizing the rudder or thruster control inputs calculated by an improved control method, e.g., model predictive control (MPC), which can reduce the energy consumption by tuning the matrices of the cost function to optimize the predictive inputs.

Nowadays, many control methods have achieved high tracking accuracy, such as sliding mode control [11,12], neural network control [13,14], and fuzzy control [15,16]. In addition, some published articles, such as [17,18,19,20], pay attention to the energy consumption optimization of AUVs by improving the control method. In [17,18], an energy suboptimizer block based on the Euler–Lagrange equation is introduced into the cost function to address the issue of energy optimization. The simulation results illustrate that the energy suboptimizer block saves a substantial amount of energy at the cost of sacrificing accuracy within an acceptable bound. A reward function is proposed to reduce energy consumption and sudden variations of the control signals when a reinforcement learning neural networks method is used for AUV control in [19]. The reward function consists of a square tracking error, an input, and input changes. The square tracking error term is used to penalize deviations of the actual trajectory from the desired trajectory, and the input and input changes terms are used to minimize the thruster use to optimize energy consumption. An improved linear quadratic regulator (LQR) is developed in [20], which chooses a cost function composed of a square tracking error and a square input. Energy consumption is reduced by optimizing the weight matrices with the genetic algorithm.

For its advantages of receding horizon optimization and handling constraints explicitly, MPC has become a mature technology in industries today [21], and many researchers implement MPC in the control of AUVs [22]. With respect to the efficient usage of resources, in [23,24], an event-based generalized predictive control (GPC) solution is developed to deal with the actuator deadband, with the core idea of tuning the control signal only when significant modification is needed to reduce frequent changes in the actuators, to reduce the resources’ utilization. In [25], an innovative hybrid agent-based MPC control method is developed, with a cost function that depends on the fuel cost, the exergy destruction cost, and the primary exergy destruction cost, to reduce energy consumption for room air temperature maintenance. A novel cost function for energy consumption, with the addition of a controlled heating/cooling power term, is defined in [26], and the efficiency of the cost function for energy consumption is illustrated by test cases. However, as far as we know, there are no results on the energy consumption optimization of AUVs with the MPC method.

The control law of MPC is constructed by minimizing the cost function. The terms of the cost function are linked to the minimization of some control objectives. Generally, these are the deviations of the predicted outputs from the reference trajectory and the predicted changes of the input vector [27,28,29]. For its attractive features of the receding horizon control principle and the ability to handle constraints explicitly [30], MPC has been implemented in the control of AUVs and remotely operated vehicles (ROVs). A simulation in [31,32,33] confirms the feasibility of MPC. Experiments with an MPC controller for AUVs or ROVs have been carried out, and the experimental results provide clear evidence to show that MPC can achieve a high control accuracy [34,35,36]. However, there is no term that optimizes the energy consumption in the cost function of MPC.

In our previous work, we have applied MPC to the depth control of AUVs together with a predictive model of a state-space model [37]. The advantage of high accuracy during trajectory tracking control makes it possible to reduce the energy consumption at the cost of compromising accuracy within an acceptable boundary. It is worthy of study for AUVs with limited energy resources.

This paper focuses on energy consumption optimization using MPC based on the state space model of AUVs. A quadratic energy term is added into the cost function of MPC to reduce the energy consumption while minimizing the deviations of the predicted outputs from the reference trajectory and the predicted changes of the input. Furthermore, a simulation with the UVIC-I AUV model [37,38] is carried out to validate the effectiveness of the proposed MPC methods.

This paper is organized as follows. Section 2 describes MPC based on an AUV’s state-space model, and an analysis of the shortcoming of energy consumption for the cost function of MPC is given. A quadratic energy term is introduced into the cost function, and the MPC control law is reconstructed in Section 3. The effectiveness of the proposed approaches is verified by simulations in Section 4. Finally, we conclude the paper in Section 5.

## 2. Problem Formulation

In this section, the conventional MPC controller is designed based on the predictive model of the discrete-time state space model of AUVs’ depth. Then, the analysis of the weakness of the control law with respect to energy consumption optimization is given.

### 2.1. MPC Control Law of AUVs’ Depth Control

According to the dynamic equation of AUVs in reference [39], the discrete-time state space model can be written as shown in Equations (1) and (2).

(1) $$x\left(k+1\right)=\left[\begin{array}{l} z\left(k+1\right)\\ v_{z}\left(k+1\right) \end{array}\right]=A\left[\begin{array}{c} z\left(k\right)\\ v_{z}\left(k\right) \end{array}\right]+Bu\left(k\right)$$

(2) $$y\left(k\right)=C\left[\begin{array}{c} z\left(k\right)\\ v_{z}\left(k\right) \end{array}\right]$$

where ***x***(*k*), *u*(*k*), and *y*(*k*) are the discrete-time state, input, and output vectors, respectively, *z*(*k*) is the depth of the AUV, *v_z_*(*k*) is the linear velocity in depth with coordinates in the body-fixed frame in the discrete-time domain, and *k* is the time in the discrete-time domain. ***A*** is the state matrix, ***B*** is the input-to-state matrix, and ***C*** is the state-to-output matrix. The specific calculation methods of ***A***, ***B***, and ***C*** are given in reference [37].

Embedding an integrator into the model to ensure zero steady-state errors, we take a difference operation on both sides of Equations (1) and (2) [28,29]. Then, Equations (1) and (2) can be expressed as Equations (3) and (4):

(3) Δx(k+1)=AΔx(k)+BΔu(k)

(4) y(k)=CΔx(k)+y(k−1)

where Δ***x***(*k*) = ***x***(*k*) − ***x***(*k* − 1) is the difference of the state, and Δ*u*(*k*) = *u*(*k*) − *u*(*k* − 1) is the difference of the input.

In most of the existing studies on predictive control, the cost function *J*_1_ has the form of Equation (5) [27,28,29,34,35,36,37].

(5) $$J_{1}={\left\|\Gamma_{y}\left(Y_{p}\left(k+1\right)-R\left(k+1\right)\right)\right\|}^{2}+{\left\|\Gamma_{u}\Delta U\left(k\right)\right\|}^{2}$$

where ***Y****_p_*(*k* + 1) is the predicted output vector with a prediction horizon of *p* at sample time *k* and ***Y****_p_*(*k* + 1) = [*y*(*k* + 1)^T^ *y*(*k* + 2)^T^ *y*(*k* + 3)^T^ … *y*(*k* + *p*)^T^]^T^, where *p* is the prediction horizon, and ***R***(*k* + 1) is the given reference vector at sample time *k* and ***R***(*k* + 1) = [*r*(*k* + 1)^T^ *r*(*k* + 2)^T^ *r*(*k* + 3)^T^ … *r*(*k* + *p*)^T^]^T^, where T represents the transpose of the matrix. **Δ*U***(*k*), the decision variable, is the predictive input changes vector at sample time *k* and **Δ*U***(*k*) = [Δ*u*(*k*)^T^ Δ*u*(*k* + 1)^T^ Δ*u*(*k* + 2)^T^ … Δ*u*(*k* + *m* − 1)^T^]^T^, where *m* is the control horizon. ***Γ**_y_* and ***Γ****_u_* are weight diagonal matrices for the predictive deviation vector and the predictive input changes vector, respectively, which are usually taken as constant matrices with compatible dimensions [29,31,34]. Setting ***Γ****_y_* = ***I****_p_*_×*p*_, where ***I****_p_*_×*p*_ is an identity matrix with a dimension of *p*, we can adjust ***Γ****_u_* according to the weights of penalizing the deviations of the predicted output ***Y****_p_*(*k* + 1) from the given trajectory ***R***(*k* + 1) and the predicted input changes **Δ*U***(*k*).

Equation (5) has an equal form as shown in Equation (6):

(6) $$J_{1}^{\prime }=\Delta U{\left(k\right)}^{T}\left(S_{u}^{T}\Gamma_{y}^{T}\Gamma_{y}S_{u}+\Gamma_{u}^{T}\Gamma_{u}\right)\Delta U\left(k\right)-2\Delta U{\left(k\right)}^{T}S_{u}^{T}\Gamma_{y}^{T}\Gamma_{y}\left[R\left(k+1\right)-S_{x}\Delta x\left(k\right)-I_{c}y\left(k\right)\right]$$

where

$$S_{u}=\left[\begin{array}{ccccc} \mathrm{CB} & 0 & 0 & \ldots & 0\\ \sum_{i=1}^{2}CA^{i-1}B & \mathrm{CB} & 0 & \ldots & 0\\ \ldots & \ldots & \ldots & \ldots & 0\\ \sum_{i=1}^{m}CA^{i-1}B & \sum_{i=1}^{m-1}CA^{i-1}B & \ldots & \ldots & \mathrm{CB}\\ \ldots & \ldots & \ldots & \ldots & \ldots \\ \sum_{i=1}^{p}CA^{i-1}B & \sum_{i=1}^{p-1}CA^{i-1}B & \ldots & \ldots & \sum_{i=1}^{p-m+1}CA^{i-1}B \end{array}\right]$$

$$S_{x}=\left[\begin{array}{c} \mathrm{CA}\\ \sum_{i=1}^{2}CA^{i}\\ \vdots \\ \sum_{i=1}^{p}CA^{i} \end{array}\right],I_{c}=\left[\begin{array}{c} 1\\ 1\\ \vdots \\ 1 \end{array}\right].$$

To simplify the following expression, we denote:

(7) $$E_{p}\left(k+1\right)=R\left(k+1\right)-S_{x}\Delta x\left(k\right)-I_{c}y\left(k\right)$$

(8) $$E^{\prime }=2\left(S_{u}^{T}\Gamma_{y}^{T}\Gamma_{y}S_{u}+\Gamma_{u}^{T}\Gamma_{u}\right)$$

(9) $$F^{\prime }=-2S_{u}^{T}\Gamma_{y}^{T}\Gamma_{y}E_{p}\left(k+1\right).$$

To minimize *J*_1_′, a routine analysis gives the solution of the predicted input changes vector as Equation (10):

(10) $$\Delta U\left(k\right)=-{E^{\prime }}^{-1}F^{\prime }.$$

### 2.2. Energy Consumption Problem for the Cost Function of MPC

As can be seen in Equation (5), the first term of the cost function penalizes deviations of the predicted outputs ***Y****p*(*k* + *1*) from the reference trajectory ***R***(*k* + *1*) to minimize the track error. According to [29,31,34,37], we set ***Γ****_y_* = ***I****_p_*_×*p*_, implying that the cost function penalizes every deviation point in the prediction horizon. The second term of Equation (5) penalizes the predicted input changes **Δ*U***(*k*) to reduce the sensitivity to environmental disturbances and small fluctuations from the sensors. According to [29,31,34], we set:

(11) $$\Gamma_{u}=\alpha \cdot I_{m\times m}$$

implying that the cost function penalizes every input change in the control horizon.

The cost function aims at the objective of minimizing its terms, such as deviations and input changes, in Equation (5). It can be seen from the action of each term in Equation (5) that there is no term for minimizing energy consumption in the cost function. For AUVs with limited onboard energy, the input changes **Δ*U***(*k*) (i.e., the control signal changes), calculated by minimizing the cost function with the form of Equation (5), are not conducive to extending the working duration of AUVs. Thus, it is necessary to add a term that represents energy consumption in the cost function of MPC.

## 3. MPC Method Considering Energy Consumption

This section addresses the construction of the control law and its stability after adding an energy consumption term to the cost function of MPC. Different from the conventional cost function in the form of Equation (5), a quadratic energy term is introduced into Equation (5) in this paper. The MPC control law is reconstructed with the new form of the cost function, and then its stability is proved.

### 3.1. The MPC Control Law Considering Energy Consumption

Consider the quadratic energy cost function [39] in Equation (12):

(12) $$J_{2}=U{\left(k\right)}^{T}\Theta U\left(k\right)$$

where ***U***(*k*) is the predictive input and ***U***(*k*) = [*u*(*k*)^T^ *u*(*k* + 1)^T^ *u*(*k* + 2)^T^ ··· *u*(*k* + *m* − 1)^T^]^T^, and ***Θ*** is the weight matrix of quadratic energy. Same to ***Γ****_y_* and ***Γ****_u_*, ***Θ*** is a positive definite matrix, usually diagonal, weighting the energy consumption. In this paper, ***Θ*** is expressed as Equation (13):

(13) $$\Theta =\beta \cdot I_{m\times m}.$$

Taking Equation (13) as a subterm, the cost function considering energy consumption *J*’ can be expressed as Equation (14):

(14) $$J^{\prime }=J_{1}+J_{2}={\left\|\Gamma_{y}\left(Y_{p}\left(k+1\right)-R\left(k+1\right)\right)\right\|}^{2}+{\left\|\Gamma_{u}\Delta U\left(k\right)\right\|}^{2}+U{\left(k\right)}^{T}\Theta U\left(k\right).$$

To find the optimal **Δ*U***(*k*) by minimizing *J*′, it is necessary to express ***U***(*k*) in the following form:

(15) $$U\left(k\right)=\left[\begin{array}{c} u\left(k\right)\\ u\left(k+1\right)\\ \vdots \\ u\left(k+m-1\right) \end{array}\right]=\left[\begin{array}{c} u\left(k-1\right)\\ u\left(k-1\right)\\ \vdots \\ u\left(k-1\right) \end{array}\right]+\left[\begin{array}{ccccc} 1 & 0 & \cdots & \cdots & 0\\ 1 & 1 & 0 & \cdots & 0\\ \vdots & \vdots & \vdots & \vdots & \vdots \\ 1 & 1 & \cdots & \cdots & 1 \end{array}\right]\Delta U\left(k\right).$$

We denote

(16) $$\overset{\leftarrow }{U}={\left[\begin{array}{cccc} u\left(k-1\right) & u\left(k-1\right) & \cdots & u\left(k-1\right) \end{array}\right]}^{T}$$

(17) $$I_{\Delta }=\left[\begin{array}{ccccc} 1 & 0 & \cdots & \cdots & 0\\ 1 & 1 & 0 & \cdots & 0\\ \vdots & \vdots & \vdots & \vdots & \vdots \\ 1 & 1 & \cdots & \cdots & 1 \end{array}\right]$$

where $\overset{\leftarrow }{U}$ is a vector of *m* × 1 dimension and ***I***_△_ is a matrix of *m* × *m* dimension. Substituting Equation (15) into Equation (12), *J*_2_ is obtained as:

(18) $$\begin{array}{ll} J_{2} & ={\left(\overset{\leftarrow }{U}+I_{\Delta }\Delta U\left(k\right)\right)}^{T}\Theta \left(\overset{\leftarrow }{U}+I_{\Delta }\Delta U\left(k\right)\right)\\ & ={\overset{\leftarrow }{U}}^{T}\Theta \overset{\leftarrow }{U}+{\overset{\leftarrow }{U}}^{T}\Theta I_{\Delta }\Delta U\left(k\right)+\Delta U{\left(k\right)}^{T}{I_{\Delta }}^{T}\Theta \overset{\leftarrow }{U}+\Delta U{\left(k\right)}^{T}{I_{\Delta }}^{T}\Theta I_{\Delta }\Delta U\left(k\right) \end{array}$$

Combining Equations (6), (8), (9), (14), and (18), and yielding to *J*, the equal form of *J*′ in Equation (14) can be expressed as Equation (19):

(19) $$\begin{array}{ll} J & =\frac{1}{2}\Delta U{\left(k\right)}^{T}E^{\prime }\Delta U\left(k\right)+\Delta U{\left(k\right)}^{T}F^{\prime }+2\Delta U{\left(k\right)}^{T}{I_{\Delta }}^{T}\Theta \overset{\leftarrow }{U}+\Delta U{\left(k\right)}^{T}{I_{\Delta }}^{T}\Theta I_{\Delta }\Delta U\left(k\right)\\ & =\frac{1}{2}\Delta U{\left(k\right)}^{T}\left(E^{\prime }+2{I_{\Delta }}^{T}\Theta I_{\Delta }\right)\Delta U\left(k\right)+\Delta U{\left(k\right)}^{T}\left(F^{\prime }+2{I_{\Delta }}^{T}\Theta \right) \end{array}$$

Like Equations (8) and (9), we denote:

(20) $$E=E^{\prime }+2{I_{\Delta }}^{T}\Theta I_{\Delta }=2\left(S_{u}^{T}\Gamma_{y}^{T}\Gamma_{y}S_{u}+\Gamma_{u}^{T}\Gamma_{u}+{I_{\Delta }}^{T}\Theta I_{\Delta }\right)$$

(21) $$F=F^{\prime }+2{I_{\Delta }}^{T}\Theta \overset{\leftarrow }{U}=-2\left(S_{u}^{T}\Gamma_{y}^{T}\Gamma_{y}E_{p}\left(k+1\right)-{I_{\Delta }}^{T}\Theta \overset{\leftarrow }{U}\right).$$

Then, the solution of the predicted input changes vector of MPC considering energy consumption is obtained as the first sub-vector of **∆*U***(*k*)

(22) $$\Delta U\left(k\right)=-E^{-1}F.$$

With the receding horizon control principle of MPC, only the first subvector of **∆*U***(*k*) is implemented in the plant, which can be calculated by Equation (23).

(23) $$\Delta u\left(k\right)=\left[\begin{array}{cccc} 1 & 0 & \cdots & 0 \end{array}\right]\cdot \Delta U\left(k\right)=K_{U\to u}\cdot \Delta U\left(k\right)=-K_{U\to u}E^{-1}F$$

where ***K****_U_*_→_*_u_* is a matrix of *m* × 1 dimension, and ***K****_U_*_→_*_u_* = [1 0 … 0]. Additionally, the actual control signal applied to the plant is:

(24) u(k)=u(k−1)+Δu(k).

### 3.2. Stability Analysis

To discuss the stability of MPC with a cost function considering quadratic energy, we substitute Equations (4), (7), (20), (21), and (22) into Equation (3), and yield to Equation:

(25) $$\begin{array}{ll} \Delta x\left(k+1\right) & =\left[A-BK_{U\to u}{\left(S_{u}^{T}\Gamma_{y}^{T}\Gamma_{y}S_{u}+\Gamma_{u}^{T}\Gamma_{u}+{I_{\Delta }}^{T}\Theta I_{\Delta }\right)}^{-1}S_{u}^{T}\Gamma_{y}^{T}\Gamma_{y}\left(S_{x}+I_{c}C\right)\right]\Delta x\left(k\right)\\ & +BK_{U\to u}{\left(S_{u}^{T}\Gamma_{y}^{T}\Gamma_{y}S_{u}+\Gamma_{u}^{T}\Gamma_{u}+{I_{\Delta }}^{T}\Theta I_{\Delta }\right)}^{-1}S_{u}^{T}\Gamma_{y}^{T}\Gamma_{y}\left[R\left(k+1\right)-I_{c}Cx\left(k-1\right)\right]\\ & -BK_{U\to u}{\left(S_{u}^{T}\Gamma_{y}^{T}\Gamma_{y}S_{u}+\Gamma_{u}^{T}\Gamma_{u}+{I_{\Delta }}^{T}\Theta I_{\Delta }\right)}^{-1}{I_{\Delta }}^{T}\Theta \overset{\leftarrow }{U} \end{array}$$

We denote the matrix ***K*_∆*x*_** by

(26) $$K_{\Delta x}=\left[A-BK_{U\to u}{\left(S_{u}^{T}\Gamma_{y}^{T}\Gamma_{y}S_{u}+\Gamma_{u}^{T}\Gamma_{u}+{I_{\Delta }}^{T}\Theta I_{\Delta }\right)}^{-1}S_{u}^{T}\Gamma_{y}^{T}\Gamma_{y}\left(S_{x}+I_{c}C\right)\right].$$

According to [40,41], if the amplitude of all eigenvalues of ***K*_∆_***_x_* is less than or equal to 1, the system is asymptotically stable. It can be seen from Equation (26) that the amplitude of eigenvalues of ***K*****_∆_***_x_* is related to ***A***, ***B***, ***C***, *p*, *m*, *α*, and *β*.

In the simulation of this paper, the dynamic model of the AUV we selected is the same as the model in [37,38], as shown in Equation (27):

(27) $$135.0727\ddot{z}+\left(214.9528-41.8985\left|\dot{z}\right|\right)\dot{z}=u.$$

In the application in reference [37], the AUV is designed for autonomous operations with a very low speed, working nearby *v_z_* = 0 m/s; thus, we linearized the plant at *v_z_* = 0 m/s. Additionally, the experimental results in [37] illustrate that the selection of *p* and *m* is appropriate for the model of Equation (27). For the selection of the same dynamic model and control method in reference [37], the matrices ***A***, ***B***, and ***C*** and the control parameters *p* and *m* selected in our paper are given in Table 1.

**Figure 1:**
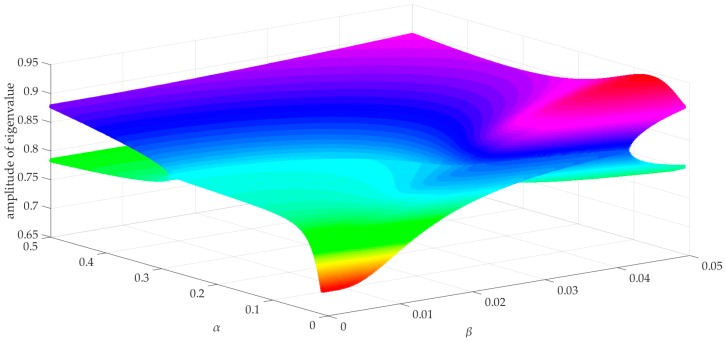
The Amplitude of All Eigenvalues of ***K*_∆_***_x_*.

Figure 1 shows the amplitude of all eigenvalues of ***K*_∆_***_x_* when *α* varies from 0.01 to 0.5 and *β* varies from 0.00 to 0.05. From Figure 1, it can be seen that the amplitude of all the eigenvalues is less than or equal to 1. It indicates that the system with a cost function considering energy consumption is asymptotically stable if *α* belongs to interval [0.01, 0.5] and *β* belongs to interval [0, 0.05].

## 4. Simulation Results and Discussion

In this section, simulation results are given to illustrate the effectiveness of the proposed method (i.e., MPC using the cost function considering energy consumption), and the comparison method is the conventional MPC (i.e., MPC using the cost function without considering energy consumption). At the end of this section, different uncertainties in system dynamics are considered, and a simulation of tracking the sinusoidal and triangular trajectory is added to demonstrate the effectiveness of the proposed method in coping with model uncertainties.

The reference trajectories in this paper are the step trajectory, the sinusoidal trajectory, and the triangular trajectory. We evaluate the simulation results with the settling time, the average of absolute tracking error, and the quadratic energy consumption, when tracking the step trajectory. The maximum of absolute tracking error, the average of absolute tracking error, and the quadratic energy consumption are used to evaluate the simulation results, when tracking the sinusoidal trajectory and the triangular trajectory. All of the values are calculated from the data in the simulation intervals.

According to [17,39], quadratic energy consumption is calculated using Equation (28):

(28) $$E_{u^{2}}=\sum_{i=1}^{i=\frac{T_{sim}}{T_{c}}}{u_{i}}^{T}\cdot u_{i}$$

and the percentage of energy consumption reduction is calculated with Equation (29):

(29) $$per=\frac{\sum_{i=1}^{i=\frac{T_{sim}}{T_{c}}}{{u^{\prime }}_{i}}^{T}\cdot {u^{\prime }}_{i}-\sum_{i=1}^{i=\frac{T_{sim}}{T_{c}}}{u_{i}}^{T}\cdot u_{i}}{\sum_{i=1}^{i=\frac{T_{sim}}{T_{c}}}{{u^{\prime }}_{i}}^{T}\cdot {u^{\prime }}_{i}}\times 100\%$$

where *T_sim_* is the simulation interval, *T_c_* is the control interval, *u_i_* is the input of MPC of this paper at time *i*, and *u_i_*′ is the input of conventional MPC at time *i*.

In the simulation, the input subjects to −16 N ≤ *u*(*k*) ≤ 16 N. The depth sensor we usually used for the AUV has a Gauss noise with an intensity of about 20–28 dBW, which is caused by an output ripple of the power depth sensor supply, circuit interference, or other reasons. Thus, in this paper, a Gauss noise with an intensity of 28 dBW, in millimeters, is added to the output to simulate interference in practice. It can be seen from Figure 1 that the amplitude of eigenvalues of ***K***_∆*x*_ increases as *α* and *β* increase. The smaller *α* and *β*, the better the system stability becomes. Thus, we choose *α* in the interval of [0, 0.05] and *β* in the interval of [0, 0.005]. To obtain an acceptable trade-off between the tracking error and energy consumption, finally we set *α* = 0.01 and *β* = 0.0001. To simulate the practice in [27] as much as possible, the control interval *T_c_* is set to 1/6 s and the simulation interval *T_sim_* is set to 400 s. The parameters for the simulation are summarized in Table 2.

### 4.1. Simulation of Tracking the Step Trajectory

In this simulation, the initial depth of the AUV is 0 m, and the initial speed is 0 m/s. The reference depth trajectory in this paper is considered as:

(30) $$z_{d}\left(t\right)=\left\{ \begin{array}{ll} 1.0 & 0\;s\leq t<200\;s\\ 0.0 & 200\;s\leq t<400\;s \end{array}\right..$$

**Figure 2:**
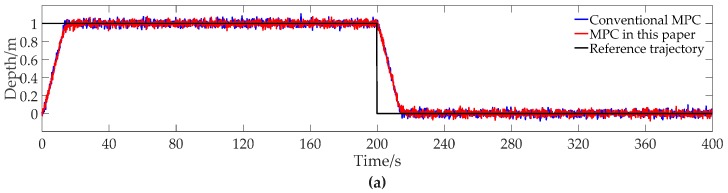

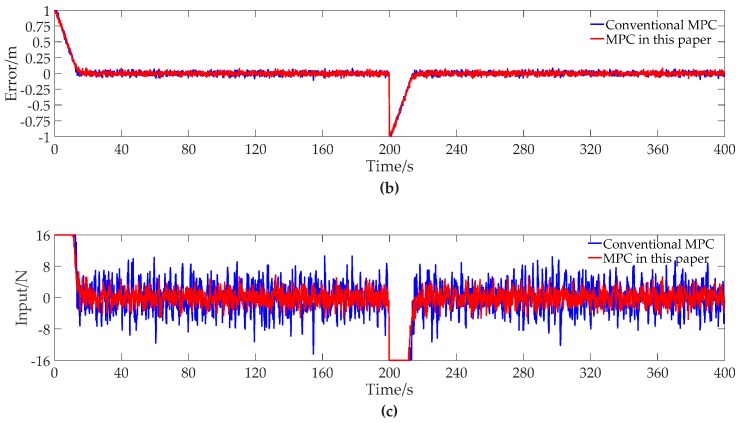
Contrast simulation results of tracking step trajectory with conventional model predictive control (MPC) and MPC of this paper. (**a**) Depth data; (**b**) Error data; (**c**) Input data.

The contrast simulation results of tracking the step trajectory with conventional MPC and MPC of this paper are shown in Figure 2.

It can be seen from Figure 2a,b that both the conventional MPC and MPC of this paper can track the reference trajectory precisely. The settling time with MPC of this paper is little longer than that with conventional MPC, changing from 12.67 s to 12.83 s. The depth simulation curves of the contrast methods are almost same in the steady stage. From Figure 2c, the input with MPC of this paper has less fluctuation than the input with conventional MPC, resulting in less energy consumption.

To give a quantitative analysis, the results of Figure 2 are summarized in Table 3. From Table 3, it can be seen that the average of absolute error over the entire simulation changes from 0.05600 m to 0.05622 m and the average of absolute error over the steady stage changes from 0.02020 m to 0.02001 m, implying that the tracking accuracy with MPC of this paper is a little worse than that with conventional MPC. However, the quadratic energy consumption reduces from 69,813 N^2^ to 46141 N^2^, with a reduction percentage of 33.91%. The data in Table 3 shows that MPC of this paper can reduce the energy consumption obviously when tracking the step trajectory.

### 4.2. Simulation of Tracking the Sinusoidal Trajectory

In this simulation, the initial depth of the AUV is 0 m, and the initial speed is 0 m/s. The reference depth trajectory in this paper is considered as:

(31) $$z_{d}\left(t\right)=\sin\left(\pi t/100\right)+1.0\;0\;s\leq t<400\;s.$$

**Figure 3:**
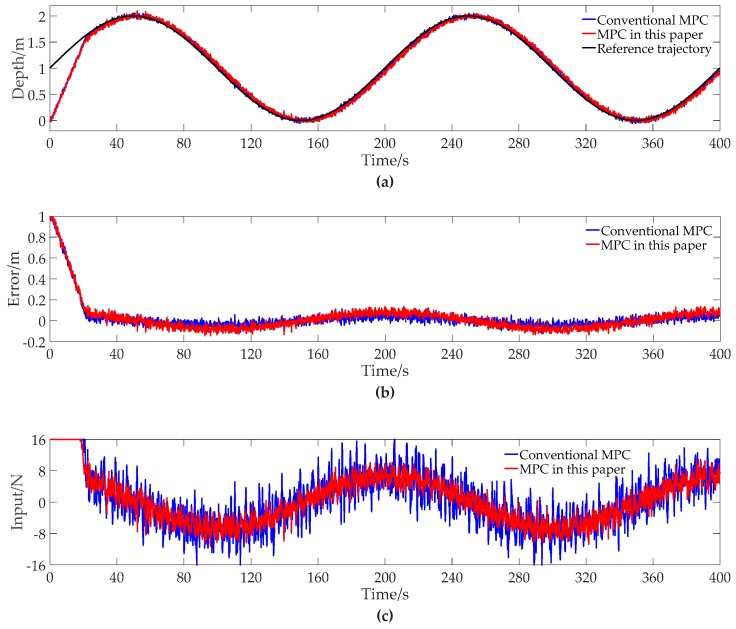
The contrast simulation results of tracking the sinusoidal trajectory with conventional MPC and MPC of this paper. (**a**) Depth data; (**b**) Error data; (**c**) Input data.

The contrast simulation results of tracking the sinusoidal trajectory with conventional MPC and MPC of this paper are shown in Figure 3.

From Figure 3a, we can see that both the conventional MPC and MPC of this paper can track the reference trajectory well, even if the initial deviation of the depth from the reference trajectory is large. It can be seen from Figure 3b that the maximum of absolute tracking error with MPC of this paper is a little larger than that with conventional MPC when the initial deviation is eliminated. Similar to tracking the step trajectory, from Figure 3c, the input with MPC of this paper has less fluctuation than the conventional MPC, leading to less energy consumption.

To give a quantitative analysis, we summarize the results of Figure 3 into Table 4. It can be seen from Table 4 that the maximum of absolute tracking error changes from 0.12526 m to 0.13748 m and the average of absolute error over the entire simulation changes from 0.03264 to 0.05479 m, meaning that the tracking accuracy with MPC of this paper is a little worse than that with conventional MPC. However, MPC of this paper cuts the quadratic energy consumption down from 113,380 N^2^ to 88,334 N^2^, with a reduction percentage of 22.09%. The data in Table 4 can illustrate that MPC of this paper is feasible for energy consumption reduction when tracking the sinusoidal trajectory.

### 4.3. Simulation of Tracking the Triangular Trajectory

In this simulation, the initial depth of the AUV is 1 m, and the initial speed is 0 m/s. The reference depth trajectory in this paper is considered as:

(32) $$z_{d}\left(t\right)=\left\{ \begin{array}{ll} 0.02t & 0\;s\leq t<100\;s\\ -0.02\left(t-100\right)+2.0 & 100\;s\leq t<200\;s\\ 0.02\left(t-200\right) & 200\;s\leq t<300\;s\\ -0.02\left(t-300\right)+2.0 & 300\;s\leq t<400\;s \end{array}\right..$$

**Figure 4:**
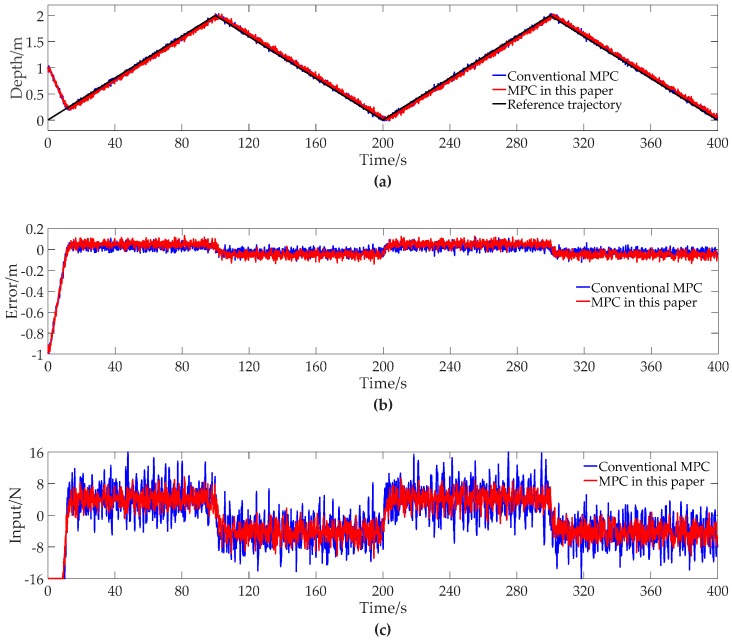
The contrast simulation results of tracking the triangular trajectory with conventional MPC and MPC of this paper. (**a**) Depth data; (**b**) Error data; (**c**) Input data.

The contrast simulation results of tracking the triangular trajectory with conventional MPC and MPC of this paper are shown in Figure 4.

Similar to the simulation of tracking the sinusoidal trajectory, in Figure 4, both the conventional MPC and MPC of this paper can track the reference trajectory well. The maximum of absolute tracking error using MPC of this paper is a little larger when the initial deviation is eliminated. In Figure 4c, the input with MPC of this paper, with less fluctuation, results in less energy consumption.

The results of Figure 4 are summarized into Table 5 for a quantitative analysis. It can be seen from Table 5 that the maximum of absolute tracking error changes from 0.12219 m to 0.14056 m and the average of absolute error over the entire simulation changes from 0.03073 to 0.05173 m. The tracking accuracy with MPC of this paper is a little worse. The quadratic energy consumption reduces from 89,207 N^2^ to 65,113 N^2^, with a reduction percentage of 27.01%. The results illustrate that MPC of this paper has the ability to reduce the energy consumption when tracking the triangular trajectory.

### 4.4. Simulation with Model Uncertainties

Similar to [42,43], we use multiplicative uncertainty in this simulation, e.g., 10% uncertainty means that the plant model is (1–10%) of the model used for control design in the continuous-time domain. Three cases (10%, 30%, and 50%) of uncertainty in system dynamics are simulated, with the same Gauss noise.

**Figure 5:**
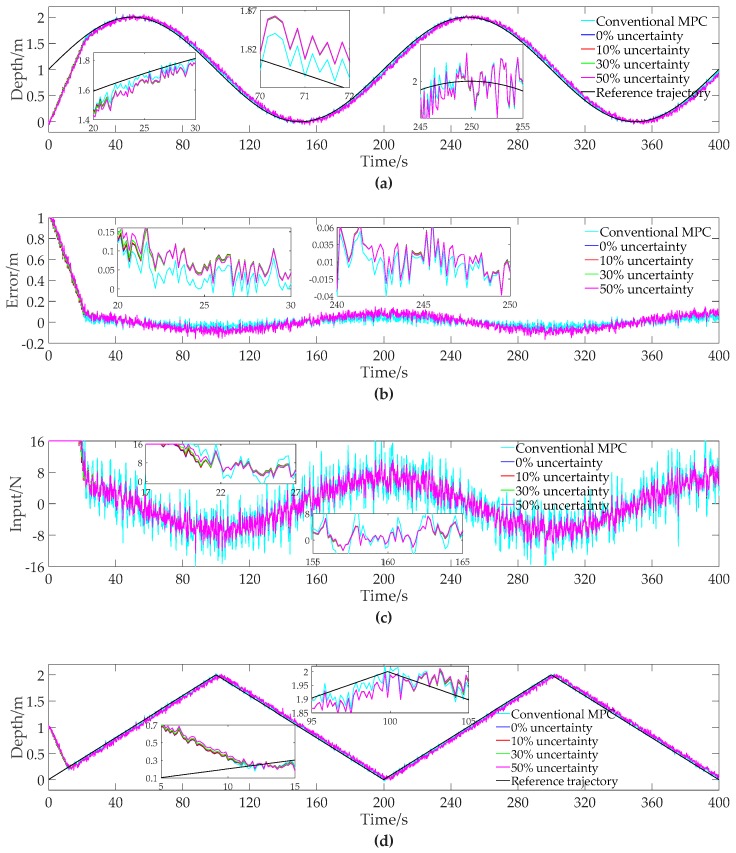

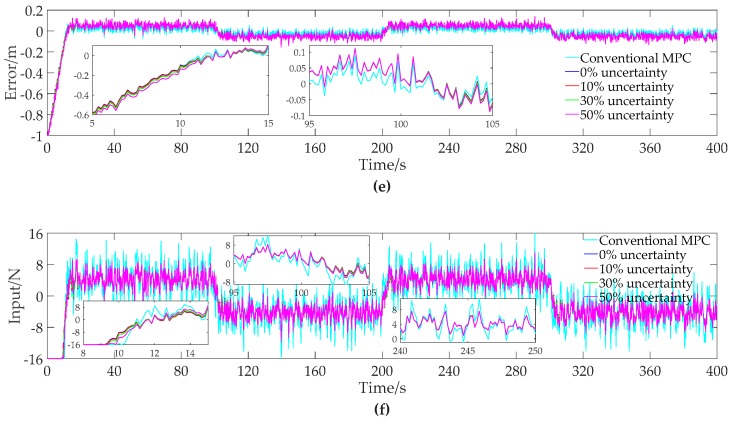
The contrast simulation results of tracking the sinusoidal and triangular trajectories with 10%, 30%, and 50% system dynamic uncertainty. (**a**) Depth data of tracking the sinusoidal trajectory; (**b**) Error data of tracking the sinusoidal trajectory; (**c**) Input data of tracking the sinusoidal trajectory; (**d**) Depth data of tracking the triangular trajectory; (**e**) Error data of tracking the triangular trajectory; (**f**) Input data of tracking the triangular trajectory.

In this simulation, the initial depth of the AUV is 0 m, and the initial speed is 0 m/s when tracking the sinusoidal trajectory. The reference depth trajectory is as shown in Equation (31). The initial depth of the AUV is 1 m, and the initial speed is 0 m/s when tracking the triangular trajectory. The reference depth trajectory is as shown in Equation (32). The results are shown in Figure 5, and the results of Figure 5 are summarized into Table 6 and Table 7.

It can be seen from Figure 5, Table 6 and Table 7 that the system dynamics uncertainty has a tiny effect on the maximum of absolute tracking error and the average of absolute tracking error, meaning that the MPC method in this paper is insensitive to the uncertainty in system dynamics on tracking accuracy. The percentage of energy consumption reduction decreases slightly with the increase of model uncertainty, whereas the reduction percentage is still more than 20%, meaning that the proposed method is feasible and effective for reducing energy consumption in AUVs even with great model uncertainty.

## 5. Conclusions

The contribution in this paper is on energy consumption reduction based on the MPC method for AUVs’ trajectory tracking control. A quadratic energy consumption term is introduced into the cost function of MPC to improve MPC with respect to energy consumption. The simulation results on an AUV provide clear evidence to show that the proposed MPC with a quadratic energy consumption term added into the cost function is feasible and effective for energy consumption reduction. The following work needs to be done in the future. Firstly, we need to investigate the rule between energy consumption and control accuracy to reduce energy consumption without sacrificing control accuracy. Secondly, a lake or sea test also needs to be conducted to validate the feasibility of the new design.

## Figures and Tables

**Table 1:** The Values of Some Matrices and Parameters Used in the Stability Analysis.

Matrices and Parameters	*A*	*B*	*C*	*p*	*m*
Value	$\left[\begin{array}{cc} 1 & 0.1464\\ 0 & 0.7670 \end{array}\right]$	$\left[\begin{array}{c} 0.0001\\ 0.0011 \end{array}\right]$	$\left[\begin{array}{cc} 1 & 0 \end{array}\right]$	80	8

**Table 2:** The parameters for the simulation.

Parameter	*p*	*m*	*α*	*β*	*T_c_*	*T_sim_*
Value	80	8	0.01	0.0001	1/6 s	400 s

**Table 3:** The Statistical Results of Figure 2.

	Conventional MPC	MPC in This Paper
Average of absolute tracking error 1	0.05600 m	0.05622 m
Average of absolute tracking error 2	0.02020 m	0.02001 m
Quadratic energy consumption	69,813 N^2^	46,141 N^2^
Percentage of energy consumption reduction	33.91%	

Note: Average of absolute tracking error 1 is calculated with the data over the entire simulation. Average of absolute tracking error 2 is calculated with the data in the intervals of 20 s to 200 s and 220 to 400 s (i.e., two periods in the steady stage).

**Table 4:** The Statistical Results of Figure 3.

	Conventional MPC	MPC in This Paper
Maximum of absolute tracking error	0.12526 m	0.13748 m
Average of absolute tracking error	0.03264 m	0.05479 m
Quadratic energy consumption	113,380 N^2^	88,334 N^2^
Percentage of energy consumption reduction	22.09%	

Note: The maximum and average of absolute tracking error are calculated with the data in the interval of 100 s to 400 s (i.e., a period in the stable tracking stage).

**Table 5:** The Statistical Results of Figure 4.

	Conventional MPC	MPC in This Paper
Maximum of absolute tracking error	0.12219 m	0.14056 m
Average of absolute tracking error	0.03073 m	0.05173 m
Quadratic energy consumption	89,207 N^2^	65,113 N^2^
Percentage of energy consumption reduction	27.01%	

Note: The maximum and average of absolute tracking error are calculated with the data in the interval of 100 s to 400 s (i.e., a period in the stable tracking stage).

**Table 6:** The Statistical Results of Figure 5a–c.

	Conventional MPC	0% Uncertainty	10% Uncertainty	30% Uncertainty	50% Uncertainty
Maximum of absolute error	0.13379 m	0.16651 m	0.16639 m	0.16602 m	0.16532 m
Average of absolute error	0.03227 m	0.05501 m	0.05502 m	0.05506 m	0.05510 m
Quadratic energy consumption	114,460 N^2^	88,881 N^2^	88,979 N^2^	89,263 N^2^	89,782 N^2^
Percentage of energy consumption reduction	--	22.35%	22.26%	22.01%	21.56%

Note: The maximum and average of absolute tracking error are calculated with the data in the interval of 100 s to 400 s (i.e., a period in the stable tracking stage).

**Table 7:** The Statistical Results of Figure 5d–f.

	Conventional MPC	0% Uncertainty	10% Uncertainty	30% Uncertainty	50% Uncertainty
Maximum of absolute error	0.13496 m	0.15562 m	0.15564 m	0.15549 m	0.15478 m
Average of absolute error	0.02998 m	0.05151 m	0.05155 m	0.05167 m	0.05190 m
Quadratic energy consumption	89,612 N^2^	64,614 N^2^	64,762 N^2^	65,204 N^2^	66,120 N^2^
Percentage of energy consumption reduction	--	27.90%	27.73%	27.24%	26.22%

Note: The maximum and average of absolute tracking error are calculated with the data in the interval of 100 s to 400 s (i.e., a period in the stable tracking stage).
